# 中性粒细胞胞外诱捕网在肺癌中的双重角色：机制探讨与治疗前景

**DOI:** 10.3779/j.issn.1009-3419.2025.101.01

**Published:** 2025-01-20

**Authors:** Chengdao LI, Dongge PENG, Wei SUN

**Affiliations:** 570311 海口，海南医科大学第二附属医院胸外二区; Department of Thoracic Surgery II, Second Affiliated Hospital of Hainan Medical University, Haikou 570311, China

**Keywords:** 中性粒细胞胞外诱捕网, 中性粒细胞, 肺肿瘤, Neutrophil extracellular traps, Neutrophil, Lung neoplasms

## Abstract

肺癌是中国居民中发病率和死亡率均较高的恶性肿瘤之一。在肿瘤微环境中，中性粒细胞所分泌的中性粒细胞胞外诱捕网（neutrophil extracellular traps, NETs）在肺癌的形成与进展过程中发挥着至关重要的作用。尽管NETs促进肿瘤发展的确切机制尚未完全阐明，但现有的研究已经揭示了它们在肿瘤生长、侵袭、转移以及癌症相关血栓形成中发挥多重作用。本文将结合近年来的研究，综述NETs在肺癌中的分子生物学机制及研究进展。

## Abstract (en)

Lung cancer is one of the most common and lethal malignancies in China. In the context of the tumor microenvironment, neutrophil extracellular traps (NETs) released by neutrophils exert a profound impact on the occurrence and progression of lung cancer. Although the exact mechanisms by which NETs promote tumor growth have not been fully elucidated, existing research has revealed their multiple roles in tumor growth, invasion, metastasis, and cancer-related thrombosis. This article will review the molecular biology mechanisms and research progress of NETs in lung cancer based on recent studies.

肺癌在全球范围内的发病率位居恶性肿瘤之首^[1]^。2022年全球新增肺癌病例近250万例，占所有癌症新发病例的12.4%，为全球最常见的癌症类型。此外，肺癌也是导致癌症相关死亡的主要原因，估计每年有180万人死亡，占所有癌症死亡病例的18.7%^[2]^。在中国，2022年恶性肿瘤流行情况^[3]^显示，在男性和女性群体中肺癌的死亡率与发病率均位列第一。肺癌的发生和发展受到多种复杂分子生物学机制的调控，这些机制与免疫细胞的相互作用密切相关。中性粒细胞胞外诱捕网（neutrophil extracellular traps, NETs）是由中性粒细胞在激活状态下所分泌的一种网状结构，主要由DNA、组蛋白和抗菌蛋白构成。中性粒细胞的主要任务是吞噬和消灭细菌^[4]^。在特定条件下，这些细胞能够通过形成NETs这一特殊机制来增强其抗菌效能。NETs在肺癌发生发展中的作用仍存在争议，本文就NETs在肺癌发展过程中的分子生物学机制及进展进行综述。

## 1 NETs的概述

### 1.1 NETs的组成成分

NETs是先天免疫中不可或缺的一种机制，也参与癌症进展，是近年来研究的新兴热点^[5]^。Brinkmann等^[6]^在2004年的研究中发现，中性粒细胞能够在细胞外形成一种由组蛋白、DNA和蛋白酶构成的网状结构，这种结构被称为NETs。NETs主要包含核DNA和颗粒蛋白质，它们共同构成一种纤维状网络结构。这种网状结构表面附着多种关键蛋白，包括髓过氧化物酶（myeloperoxidase, MPO）、中性粒细胞弹性蛋白酶（neutrophil elastase, NE）、白细胞蛋白酶3以及乳铁蛋白（lactoferrin, LTF）等。这些蛋白质赋予NETs多样的生物活性，使其在免疫防御和病原体清除中发挥关键作用。经高分辨率扫描电子显微镜观察，NETs主要由直径为15-17 nm的纤维以及直径为25-50 nm的球形聚集复合体组成。

### 1.2 NETs的形成过程（NETosis）

NETosis是一种涉及细胞程序性死亡的复杂生物过程，其中包括自杀性NETs（suicidal NETs）和重要性NETs（vital NETs）两种类型。NETosis启动时，细胞骨架和内膜系统解体，质膜微囊泡脱落释放颗粒和细胞质。微管和波形蛋白骨架解体重塑，内质网小泡化，染色质解凝集和核变圆，细胞膜和核膜通透性增加，核膜网和核膜破裂释放染色质，细胞膜破裂释放细胞外DNA，完成NETs形成^[7]^。这一细胞程序性死亡损伤中性粒细胞本身，但有效地释放了NETs，以对抗入侵的病原体。目前，NETosis包括NADPH依赖性和非NADPH依赖性两种途径^[8]^。在NADPH依赖性NETosis中，NADPH氧化酶激活产生活性氧簇，促使NE和MPO转移到细胞核，其中NE降解组蛋白，MPO促进此过程，肽基精氨酸脱亚胺酶4（peptidylarginine deiminase 4, PAD4）介导的组蛋白H3瓜氨酸化作用能够促使染色质结构发生解旋，从而影响基因表达和细胞功能。最终，核膜破裂，染色质与抗菌因子结合，通过细胞膜破裂释放形成NETs^[9]^。相比之下，非NADPH依赖性NETosis中，中性粒细胞保持活性，NETs的形成不涉及核膜和细胞膜的破裂，而是由细菌、细菌产物、细胞因子或化学试剂等诱导，染色质解旋和NE的核转移过程与NADPH依赖性NETosis相似，NETs以囊泡形式排出，细胞膜保持完整，中性粒细胞维持活性^[10]^。在肺癌的研究中，NETs水平的升高与肿瘤预后相关，NADPH依赖性NETosis中NETs作为生物标志物，而非NADPH依赖性NETosis中肿瘤微环境中的细胞因子如白细胞介素 8（interleukin-8, IL-8）、粒细胞集落刺激因子（granulocyte colony-stimulating factor, G-CSF）促进NETs形成，影响肿瘤的增殖和转移。因此，监测NETs水平对于评估肺癌的进展和预后具有重要临床意义^[11]^。

## 2 NETs与肺癌进展中的作用

### 2.1 NETs在肺癌肿瘤微环境中的表达

肿瘤微环境形成了一个错综复杂的网络体系，它由多种细胞类型和非细胞组分构成。在细胞层面，涉及一些关键细胞类型，包括成纤维细胞、间充质干细胞、免疫细胞以及内皮细胞等。而非细胞组分则包括细胞外基质、基质金属蛋白酶家族成员以及一系列细胞因子。这些成分通过精细的调控机制相互作用，对肿瘤细胞的生长、免疫逃逸、局部侵袭和远处转移等关键生物学过程发挥着至关重要的作用^[12]^。NETs的形成是一个可能由肿瘤微环境驱动的复杂过程。肿瘤细胞可通过分泌细胞因子直接诱导NETs的形成，同时由肿瘤细胞释放的外泌体也能刺激NETs的释放^[13]^。此外，肿瘤微环境中的炎症介质和缺氧条件进一步促进NETs的产生^[14]^。肿瘤细胞与中性粒细胞之间的相互作用，也参与了NETs的形成。巨噬细胞及其外泌体miR-146a在调节NETs水平中发挥作用，可能通过形成巨噬细胞胞外诱捕网（macrophage extracellular traps, METs）与NETs协同作用，共同影响肿瘤的迁移和侵袭^[15]^。这些相互作用的结果使NETs在肺癌中的形成增加，进而影响肿瘤的增殖和转移。在肺癌的发展过程中，癌细胞外的RNA以及由肺癌细胞释放的高迁移率族蛋白B1（high mobility group protein B1, HMGB1）在促进NETs的形成中起着关键作用^[16]^。NETs能够捕获肿瘤细胞，并在巨噬细胞所维持的炎症性微环境中，促进这些肿瘤细胞的生长、侵袭性和迁移能力^[17]^。

有证据^[18]^表明，肿瘤细胞及其微环境能够激活中性粒细胞，导致多种肿瘤类型中NETs的产生，并且这种胞外陷阱的存在与肿瘤患者较差的临床预后有关。癌细胞能够通过激活转化生长因子-β（transforming growth factor-β, TGF-β）信号传导途径来发挥作用，推动中性粒细胞从N1型向N2型的转变，这种转变使得N2型中性粒细胞的生成数量显著增加，还破坏了中性粒细胞的平衡状态。这种失衡进一步刺激了肿瘤细胞的侵袭能力^[19]^。Fridlender等^[20]^发现，中性粒细胞在肿瘤微环境中的作用远比我们预想的要复杂。N1型中性粒细胞的一个显著特点不是促进肿瘤生长，而是抑制肿瘤生长，而N2型中性粒细胞则可能促进肿瘤的进展。有研究^[21,22]^指出，具有超节状核特征的中性粒细胞群体表现出抗肿瘤的活性。

NETs在肺癌微环境中发挥着双重作用，既有可能抑制肿瘤生长，也可能促进肿瘤进展。NETs通过激活树突状细胞和加剧Th1反应参与免疫调节，有助于阻止肿瘤生长^[23]^。DNA酶治疗能够减少NETs，抑制肿瘤细胞的黏附和侵袭^[24]^。在免疫治疗中，NETs还能通过募集免疫细胞和诱导细胞凋亡来预防肿瘤增长^[25]^。然而，NETs在肺癌中通过形成物理屏障、调节免疫微环境、释放蛋白降解酶、捕获循环肿瘤细胞、改变血管完整性以及激活肿瘤细胞生长信号，对肿瘤的生长和转移发挥着促进作用^[26]^。NETs既可以作为抗肿瘤的免疫调节因子，也可以作为促进肿瘤进展和转移的关键因素。因此，NETs及其相关机制的研究对于开发新的癌症治疗策略具有重要意义。

### 2.2 NETs参与肺癌转移

NETs与肺癌的不同进展阶段，包括增殖、侵袭、转移以及免疫逃逸等方面，存在着密切的联系。在肺癌的增殖阶段，Albrengues等^[27]^通过实验发现，NETs的DNA框架与细胞外基质中的粘连蛋白相互作用，这一过程在慢性肺部炎症环境下尤为显著，如由烟草烟雾或脂多糖引发的情况。与NETs相关的NE和基质金属蛋白酶（matrix metalloproteinase, MMP）-9能够降解粘连蛋白，进而激活整合素α3β1，触发肿瘤细胞内FAK/ERK/MLCK/YAP信号通路的活化，最终导致肿瘤细胞增殖的加速。而阻断肿瘤细胞及NETs中的β1整合素，能有效减少它们之间的黏附，进而减少肺癌细胞的转移能力^[28]^。其次，在肺癌细胞的侵袭和转移过程中，NETs同样起到了关键作用。Lee等^[29]^在探究NETs在肺癌侵袭和转移过程中的作用，通过构建体外实验模型并采用数学方法模拟肿瘤细胞的侵袭行为，发现肿瘤细胞分泌的转化生长因子-β（transforming growth factor-β, TGF-β）可诱导中性粒细胞由抗肿瘤的N1表型向促肿瘤的N2表型转变。N2表型中性粒细胞分泌的NE和MMPs显著增强了肿瘤细胞的侵袭能力。此外，使用脱氧核糖核酸酶I（deoxyribonuclease I, DNase I）处理能够降低NE和NETs的活性，进而抑制肿瘤细胞的侵袭性和转移能力。

NETs在肺癌细胞的免疫逃逸过程中也扮演着关键角色。Dimitrov等^[30]^发现NETs在癌症细胞的黏附和激活Notch 1介导的上皮-间质转化（epithelial-to-mesenchymal transition, EMT）中扮演着重要角色，这些过程对于肺癌细胞的迁移和侵袭能力至关重要。NETs作为癌症细胞黏附的基质，显著增强了癌症细胞的黏附能力，而长期暴露于NETs中的癌症细胞会激活EMT程序，从而增强其迁移能力。Wang等^[31]^研究揭示，在非小细胞肺癌（non-small cell lung cancer, NSCLC）的转移过程中，NETs通过抑制长链非编码RNA（long non-coding RNA, lncRNA）MIR503HG的表达，激活了核因子κB（nuclear factor kappa-B, NF-κB）和NLRP3信号通路，从而促进了肿瘤细胞的侵袭和转移。该研究首次证明了NETs在激活炎症小体过程中对癌症扩散的影响。NETs促进了肺癌细胞的增殖、侵袭和转移、免疫逃逸，这为肺癌治疗提供了新的视角，即通过抑制NETs的形成或活性来控制肺癌的发展。

## 3 NETs在肺癌治疗中的潜在应用

### 3.1 NETs作为诊断标志物的潜力

在肺癌的研究^[32]^中，NETs在NSCLC发展中扮演复杂角色，并为未来的治疗策略提供了新的思路。在肺癌诊断领域中，NETs的检测方法多样，各有其优势与局限。采用酶联免疫吸附试验（enzyme-linked immunosorbent assay, ELISA）检测MPO-DNA等NETs重要组分的水平，虽然成本低且可自动化，但重复性差^[33]^。流式细胞术以其操作简便和高精准度受到青睐，能够快速分选亚细胞及细胞内颗粒物，但主要检测细胞质内的MPO，可能影响NETs的准确定量。荧光染色法和免疫组化方法能提供清晰的NETs成像，有助于直观观察其结构和分布，但操作复杂性限制了它们的临床应用^[34]^。基因表达分析可以提供全面的基因表达视图，而蛋白质表达检测可以提供具体的蛋白质水平信息，lncRNA分析则可以提供预后相关的lncRNA信息。

苏莹等^[35]^发现与癌旁组织相比，NSCLC患者肿瘤组织中PAD4和精氨酸酶-1（arginase-1, Arg-1）的表达水平升高。在M1型巨噬细胞极化过程中，加入NETs后，巨噬细胞中诱导型一氧化氮合酶（inducible nitric oxide synthase, iNOS）、IL-6及肿瘤坏死因子-α（tumor necrosis factor-α, TNF-α）的表达水平降低，而Arg-1、IL-10、TGF-β的表达水平升高。在M2型巨噬细胞极化过程中，加入NETs后，巨噬细胞中Arg-1、IL-10、TGF-β的表达水平升高。与对照组相比，M2组A549细胞的增殖能力、侵袭性以及VEGF表达水平显著增强。而与M2组相比，M2+NETs组A549细胞的增殖能力、侵袭性以及血管内皮生长因子（vascular endothelial growth factor, VEGF）表达水平进一步提高。NETs通过诱导巨噬细胞向M2型极化，进而增强了NSCLC细胞的增殖与侵袭能力。Zuo等^[36]^的研究通过共识聚类分析在肺腺癌（lung adenocarcinoma, LUAD）中鉴定出两个与NETs相关的分子亚型，并发现高NETs评分与较好的总生存率、丰富的免疫细胞浸润以及高活性的免疫反应信号通路存在关联。基于NETs评分，鉴定出6个具有预后价值的基因，包括*GOS2*、*KCNJ15*、*S100A12*、*AKT2*、*CTSG*和*HMGB1*，构建了预后特征，这些特征在训练集和验证集中均能显著预测低风险LUAD患者的总生存率。

肺腺癌患者的独立预后因素包括NETs相关的风险评分和临床分期，这两者在预测患者预后方面具有重要意义，且与肿瘤免疫微环境显著相关。Mo等^[37]^通过分析TCGA LUAD数据集，鉴定出6个新的NETs相关基因：*UPK1B*、*SFTA3*、*GGTLC1*、*SCGB3A1*、*ABCC2*和*NTS*。基于这些基因，研究者开发了一个NETs评分特征，该特征与LUAD患者的临床病理和免疫特征显著相关。此外，高风险患者表现出免疫相关过程的抑制，而不同群体之间的突变模式存在变异性。Ding等^[38]^通过单变量*Cox*回归，筛选了10个与LUAD患者预后有关的NETs相关lncRNAs，并发现它们与肿瘤微环境显著相关。*LASSO*回归构建了一个基于4个lncRNAs（SIRLNT、AL365181.3、FAM83A-AS1、AJ003147.2）的预后模型。*Kaplan-Meier*分析显示风险模型与训练组和验证组1和2中患者的生存结果密切相关（*P*<0.001, *P*=0.026, *P*<0.01）。NETs在NSCLC发展中起着复杂而重要的作用，随着检测技术的不断发展和完善，NETs作为诊断标志物的潜力将可能得到进一步挖掘和利用。

### 3.2 基于NETs的靶向治疗策略

中性粒细胞释放的NETs在肿瘤微环境中扮演着复杂的角色。NETs可以作为治疗靶点，并且与癌症的临床结果密切相关^[39,40]^。它们不仅构成了一道屏障，肿瘤细胞可以抵抗细胞毒性T细胞的攻击，进而实现存活^[41]^，而且还可能触发原本休眠的癌细胞重新进入活跃状态^[27]^。随着对NETs在恶性肿瘤发展中作用的深入认识，NETs作为肺癌治疗的创新靶点策略的潜力逐渐显现^[42]^（表1）。有研究^[43]^表明，编码L-DNase II的基因是*SERPINB1*，可降解NETs相关DNA，从而在一定程度上阻止NETs的合成。通过体外和体内实验研究了*SERPINB1*对NETosis的调节作用，发现*SERPINB1*缺失的小鼠骨髓和肺中性粒细胞对多种介质诱导的NETosis表现出高度敏感性，这表明*SERPINB1*在NETosis中扮演着深层的调节角色。Hao等^[44]^开发了一种新型纳米系统（DMMnSiO-PEG/DOX/DNase-1），通过递送阿霉素（Doxorubicin, DOX）和DNase-1来实现其目标，经PEG修饰的纳米载体在肿瘤组织中富集，并容易被NETs捕获。这表明PEG修饰可能促进了纳米载体与NETs之间的相互作用。在肿瘤细胞附近，纳米系统表现出pH响应行为，并对谷胱甘肽水平升高做出反应，从而促进DOX和DNase-1的释放。DNase-1可通过降解肿瘤细胞外的DNA，减少肿瘤微环境中的DNA积累，从而增强化疗药物DOX的治疗效果。包埋在NETs中的纳米系统颗粒能够分解NETs内的DNA。释放的Mn²^+^作为DNase-1的辅助因子，从而加剧了NETs的降解。另外，二甲双胍显示出对由HMGB1触发的NETosis的抑制效果，有助于减少肺癌模型小鼠体内循环NETs的水平和肿瘤免疫反应的阳性比率，从而突显了二甲双胍作为抗癌候选药物的潜力^[45]^。这些研究的最新进展揭示了NETs不仅参与肿瘤的免疫逃逸机制，还可能直接促进肿瘤细胞的活化和扩散^[5]^。

**表1:** NETs的靶向治疗

Target	Drug	Clinical research phase
SERPINB1	L-DNase II	Clinical research phase
HMGB1	Metformin	Clinical research phase
DNase 1	Inhaled DNase I formulation (Pulmozyme)	FDA approved for the treatment of CF
Mn² with DNase-1	Nanosystem particles embedded in NETs	Research phase, for the degradation of NETs

SERPINB1: serine protease inhibitor, Kazal type 1; L-DNase II: recombinant human DNase II; HMGB1: high mobility group box 1; DNase I: deoxyribonuclease I; FDA: Food and Drug Administration; CF: cystic fibrosis; NETs: neutrophil extracellular traps.

综上所述，NETs在肺癌中具有双重作用，既能促进肿瘤生长、侵袭、迁移，也能抑制肿瘤。NETs通过TGF-β信号通路促使中性粒细胞从N1型转变为N2型，增强肿瘤的侵袭性。NETs可激活信号通路，促进肿瘤细胞增殖和癌症细胞黏附、EMT，增强迁移能力；也可促进肿瘤细胞凋亡，以及调节免疫细胞，尤其是T细胞的活性，抑制肿瘤细胞的增殖。NETs在肺癌中的双重作用机制、与肿瘤微环境的相互作用、作为生物标志物和治疗靶点的潜力等，尚需深入研究。

## References

[1] Siegel RL, Giaquinto AN, Jemal A. Cancer statistics, 2024. CA Cancer J Clin, 2024, 74(1): 12-49. doi: 10.3322/caac.21820 38230766

[2] Bray F, Laversanne M, Sung H, et al. Global cancer statistics 2022: GLOBOCAN estimates of incidence and mortality worldwide for 36 cancers in 185 countries. CA Cancer J Clin, 2024, 74(3): 229-263. doi: 10.3322/caac.21834 38572751

[3] Zheng RS, Chen R, Han BF, et al. Cancer incidence and mortality in China, 2022. Zhonghua Zhongliu Zazhi, 2024, 46(3): 221-231. 10.3760/cma.j.cn112152-20240119-00035 38468501

[4] Rayes RF, Vourtzoumis P, Bou Rjeily M, et al. Neutrophil extracellular trap-associated CEACAM1 as a putative therapeutic target to prevent metastatic progression of colon carcinoma. J Immunol, 2020, 204(8): 2285-2294. doi: 10.4049/jimmunol.1900240 32169849 PMC7534954

[5] Demkow U. Neutrophil extracellular traps (NETs) in cancer invasion, evasion and metastasis. Cancers, 2021, 13(17): 4495. doi: 10.3390/cancers13174495 34503307 PMC8431228

[6] Brinkmann V, Reichard U, Goosmann C, et al. Neutrophil extracellular traps kill bacteria. Science, 2004, 303(5663): 1532-1535. doi: 10.1126/science.1092385 15001782

[7] Thiam HR, Wong SL, Qiu R, et al. NETosis proceeds by cytoskeleton and endomembrane disassembly and PAD4-mediated chromatin decondensation and nuclear envelope rupture. Proc Natl Acad Sci U S A, 2020, 117(13): 7326-7337. doi: 10.1073/pnas.1909546117 32170015 PMC7132277

[8] Chen Y, Hu H, Tan S, et al. The role of neutrophil extracellular traps in cancer progression, metastasis and therapy. Exp Hematol Oncol, 2022, 11(1): 99. doi: 10.1186/s40164-022-00345-3 36384979 PMC9667637

[9] Tokuhiro T, Ishikawa A, Sato H, et al. Oxidized phospholipids and neutrophil elastase coordinately play critical roles in NET formation. Front Cell Dev Biol, 2021, 9: 718586. doi: 10.3389/fcell.2021.718586 34568331 PMC8458647

[10] Eghbalzadeh K, Georgi L, Louis T, et al. Compromised anti-inflammatory action of neutrophil extracellular traps in PAD4-deficient mice contributes to aggravated acute inflammation after myocardial infarction. Front Immunol, 2019, 10: 2313. doi: 10.3389/fimmu.2019.02313 31632398 PMC6779806

[11] Fang C, Liu F, Wang Y, et al. A innovative prognostic symbol based on neutrophil extracellular traps (NETs)-related lncRNA signature in non-small-cell lung cancer. Aging (Albany NY), 2021, 13(13): 17864-17879. doi: 10.18632/aging.203289 34257164 PMC8312458

[12] Xiao Y, Yu D. Tumor microenvironment as a therapeutic target in cancer. Pharmacol Ther, 2021, 221: 107753. doi: 10.1016/j.pharmthera.2020.107753 33259885 PMC8084948

[13] Li J, Chen J, Sun J, et al. The formation of NETs and their mechanism of promoting tumor metastasis. J Oncol, 2023: 7022337. doi: 10.1155/2023/7022337 36942262 PMC10024627

[14] Wu DH, Zhang SG, Liu LX. The role of neutrophil extracellular traps in the tumor progression. Zhongguo Kexue Jishu Daxue Xuebao, 2018, 48(10): 781-784.

[15] Yue PP, Guo DL, Liu PP, et al. The research progress of macrophage extracellular traps. Zhonghua Shiyan Waike Zazhi, 2020, 37(10): 1964-1968.

[16] Kwak SB, Kim SJ, Kim J, et al. Tumor regionalization after surgery: roles of the tumor microenvironment and neutrophil extracellular traps. Exp Mol Med, 2022, 54(6): 720-729. doi: 10.1038/s12276-022-00784-2 35764882 PMC9256747

[17] Zhang L, Yi H, Chen J, et al. Neutrophil extracellular traps facilitate A 549 cell invasion and migration in a macrophage-maintained inflammatory microenvironment. Biomed Res Int, 2022: 8316525. doi: 10.1155/2022/8316525 PMC8758275 35036439

[18] Cristinziano L, Modestino L, Antonelli A, et al. Neutrophil extracellular traps in cancer. Semin Cancer Biol, 2022, 79: 91-104. doi: 10.1016/j.semcancer.2021.07.011 34280576

[19] Ralph SJ, Reynolds MJ. Intratumoral pro-oxidants promote cancer immunotherapy by recruiting and reprogramming neutrophils to eliminate tumors. Cancer Immunol Immunother, 2023, 72(3): 527-542. doi: 10.1007/s00262-022-03248-8 36066649 PMC9446783

[20] Fridlender ZG, Sun J, Kim S, et al. Polarization of tumor-associated neutrophil phenotype by TGF-β: “N1” versus “N2” TAN. Cancer Cell, 2009, 16(3): 183-194. doi: 10.1016/j.ccr.2009.06.017 19732719 PMC2754404

[21] Shaul ME, Fridlender ZG. Cancer-related circulating and tumor-associated neutrophils-subtypes, sources and function. FEBS J, 2018, 285(23): 4316-4342. doi: 10.1111/febs.14524 29851227

[22] Millrud CR, Kagedal A, Kumlien Georen S, et al. NET-producing CD16^high^ CD62L^dim^ neutrophils migrate to tumor sites and predict improved survival in patients with HNSCC. Int J Cancer, 2017, 140(11): 2557-2567. doi: 10.1002/ijc.30671 28247912

[23] Fang Q, Stehr AM, Naschberger E, et al. No NETs no TIME: crosstalk between neutrophil extracellular traps and the tumor immune microenvironment. Front Immunol, 2022, 13: 1075260. doi: 10.3389/fimmu.2022.1075260 36618417 PMC9816414

[24] Zhang XW, Wang Y, Li Y. Research progress of neutrophil extracellular traps in tumor metastasis. Zhongguo Feiai Zazhi, 2019, 22(9): 600-604. 31526465 10.3779/j.issn.1009-3419.2019.09.08 PMC6754575

[25] Meier P, Legrand AJ, Adam D, et al. Immunogenic cell death in cancer: targeting necroptosis to induce antitumour immunity. Nat Rev Cancer, 2024, 24(5): 299-315. doi: 10.1038/s41568-024-00674-x 38454135

[26] Yan M, Gu Y, Sun H, et al. Neutrophil extracellular traps in tumor progression and immunotherapy. Front Immunol, 2023, 14: 1135086. doi: 10.3389/fimmu.2023.1135086 36993957 PMC10040667

[27] Albrengues J, Shields MA, Ng D, et al. Neutrophil extracellular traps produced during inflammation awaken dormant cancer cells in mice. Science, 2018, 361(6409): eaao4227. doi: 10.1126/science.aao4227 30262472 PMC6777850

[28] Liu Y, Liu L. The pro-tumor effect and the anti-tumor effect of neutrophils extracellular traps. Biosci Trends, 2019, 13(6): 469-475. doi: 10.5582/bst.2019.01326 31866615

[29] Lee J, Lee D, Lawler S, et al. Role of neutrophil extracellular traps in regulation of lung cancer invasion and metastasis: structural insights from a computational model. PLoS Comput Biol, 2021, 17(2): e1008257. doi: 10.1371/journal.pcbi.1008257 33596197 PMC7920364

[30] Dimitrov J, Maddalena M, Terlizzi C, et al. Dynamic roles of neutrophil extracellular traps in cancer cell adhesion and activation of Notch 1-mediated epithelial-to-mesenchymal transition in EGFR-driven lung cancer cells. Front Immunol, 2024, 15: 1470620. doi: 10.3389/fimmu.2024.1470620 39430758 PMC11487346

[31] Wang Y, Liu F, Chen L, et al. Neutrophil extracellular traps (NETs) promote non-small cell lung cancer metastasis by suppressing lncRNA MIR503HG to activate the NF-κB/NLRP 3 inflammasome pathway. Front Immunol, 2022, 13: 867516. doi: 10.3389/fimmu.2022.867516 35707534 PMC9190762

[32] Mutua V, Gershwin LJ. A review of neutrophil extracellular traps (NETs) in disease: potential anti-NETs therapeutics. Clin Rev Allergy Immunol, 2021, 61(2): 194-211. doi: 10.1007/s12016-020-08804-7 32740860 PMC7395212

[33] Hayden H, Ibrahim N, Klopf J, et al. ELISA detection of MPO-DNA complexes in human plasma is error-prone and yields limited information on neutrophil extracellular traps formed *in vivo*. PLoS One, 2021, 16(4): e0250265. doi: 10.1371/journal.pone.0250265 33886636 PMC8062102

[34] Wang YT, Gu B, Li HN, et al. Review of detection methods for neutrophil extracellular traps (NETs). Zhongguo Yaolixue Tongbao, 2019, 35(12): 1646-1649.

[35] Su Y, Ma LL, Liu J, et al. Neutrophil extracellular traps promote proliferation and invasion of NSCLC by inducing M2-type polarization of macrophages. Zhongguo Mianyixue Zazhi, 2023, 39(3): 472-477.

[36] Zuo Y, Leng G, Leng P. Identification and validation of molecular subtype and prognostic signature for lung adenocarcinoma based on neutrophil extracellular traps. Pathol Oncol Res, 2023, 29: 1610899. doi: 10.3389/pore.2023.1610899 37143472 PMC10151567

[37] Mo G, Long X, Cao L, et al. A six-gene prognostic model based on neutrophil extracellular traps (NETs)-related gene signature for lung adenocarcinoma. Comb Chem High Throughput Screen, 2024, 27(13): 1969-1983. doi: 10.2174/0113862073282003240119064337 38357943

[38] Ding W, Li B, Zhang Y, et al. A neutrophil extracellular traps-associated lncRNA signature predicts the clinical outcomes in patients with lung adenocarcinoma. Front Genet, 2022, 13: 1047231. doi: 10.3389/fgene.2022.1047231 36419832 PMC9676361

[39] Zhang Y, Guo L, Dai Q, et al. A signature for pan-cancer prognosis based on neutrophil extracellular traps. J Immunother Cancer, 2022, 10(6): e004210. doi: 10.1136/jitc-2021-004210 35688556 PMC9189842

[40] Yang D, Liu J. Neutrophil extracellular traps: a new player in cancer metastasis and therapeutic target. J Exp Clin Cancer Res, 2021, 40(1): 233. doi: 10.1186/s13046-021-02013-6 34271947 PMC8283906

[41] Ireland AS, Oliver TG. Neutrophils create an ImpeNETrable shield between tumor and cytotoxic immune cells. Immunity, 2020, 52(5): 729-731. doi: 10.1016/j.immuni.2020.04.009 32433945 PMC7851833

[42] Shao BZ, Yao Y, Li JP, et al. The role of neutrophil extracellular traps in cancer. Front Oncol, 2021, 11: 714357. doi: 10.3389/fonc.2021.714357 34476216 PMC8406742

[43] Demkow U. Molecular mechanisms of neutrophil extracellular trap (NETs) degradation. Int J Mol Sci, 2023, 24(5): 4896. doi: 10.3390/ijms24054896 36902325 PMC10002918

[44] Hao Y, Li X, Liu Y, et al. Manganese doped nanosystem for degrading neutrophil extracellular traps and improving chemotherapy efficiency to synergistically inhibit lung metastasis of breast cancer. Chem Eng J, 2023, 466: 142957. doi: 10.1016/j.cej.2023.142957

[45] Chen CJ, Wu CC, Chang CY, et al. Metformin mitigated obesity-driven cancer aggressiveness in tumor-bearing mice. Int J Mol Sci, 2022, 23(16): 9134. doi: 10.3390/ijms23169134 36012397 PMC9408975

